# Phenotypic screening reveals TNFR2 as a promising target for cancer immunotherapy

**DOI:** 10.18632/oncotarget.11943

**Published:** 2016-09-10

**Authors:** Geoffrey S. Williams, Bina Mistry, Sandrine Guillard, Jane Coates Ulrichsen, Alan M. Sandercock, Jun Wang, Andrea González-Muñoz, Julie Parmentier, Chelsea Black, Jo Soden, Jim Freeth, Jelena Jovanović, Rebecca Leyland, Rafia S. Al-Lamki, Andrew J. Leishman, Steven J. Rust, Ross Stewart, Lutz Jermutus, John R. Bradley, Vahe Bedian, Viia Valge-Archer, Ralph Minter, Robert W. Wilkinson

**Affiliations:** ^1^ MedImmune Ltd., Granta Park, Cambridge, CB21 6GH, UK; ^2^ Department of Medicine, University of Cambridge, Addenbrooke's Hospital, Cambridge, CB2 0QQ, UK; ^3^ Oncology iMED, AstraZeneca-R&D Boston, Waltham, MA 02451, USA; ^4^ MedImmune LLC, Gaithersburg, MD 20878, USA; ^5^ Retrogenix Ltd, Whaley Bridge, High Peak, SK23 7LY, UK

**Keywords:** TNFR2, regulatory T cell, cancer immunotherapy, drug discovery, phenotypic screening

## Abstract

Antibodies that target cell-surface molecules on T cells can enhance anti-tumor immune responses, resulting in sustained immune-mediated control of cancer. We set out to find new cancer immunotherapy targets by phenotypic screening on human regulatory T (Treg) cells and report the discovery of novel activators of tumor necrosis factor receptor 2 (TNFR2) and a potential role for this target in immunotherapy. A diverse phage display library was screened to find antibody mimetics with preferential binding to Treg cells, the most Treg-selective of which were all, without exception, found to bind specifically to TNFR2. A subset of these TNFR2 binders were found to agonise the receptor, inducing iκ-B degradation and NF-κB pathway signalling *in vitro*. TNFR2 was found to be expressed by tumor-infiltrating Treg cells, and to a lesser extent Teff cells, from three lung cancer patients, and a similar pattern was also observed in mice implanted with CT26 syngeneic tumors. In such animals, TNFR2-specific agonists inhibited tumor growth, enhanced tumor infiltration by CD8^+^ T cells and increased CD8^+^ T cell IFN-γ synthesis. Together, these data indicate a novel mechanism for TNF-α-independent TNFR2 agonism in cancer immunotherapy, and demonstrate the utility of target-agnostic screening in highlighting important targets during drug discovery.

## INTRODUCTION

Immune-mediated cancer therapies, particularly monoclonal antibodies (mAbs) that target cell-surface receptors expressed by T cells, are rapidly emerging as a valuable class of cancer drugs. Clinical data indicate that antagonists of cytotoxic T lymphocyte antigen 4 (CTLA-4) and programmed cell death protein 1/programmed death-ligand 1 (PD-1/PD-L1) can lead to durable anti-tumor immune responses [1–5], and have led to widespread interest in targeting other T cell surface antigens, including co-stimulatory members of the tumor necrosis factor receptor superfamily (TNFRSF) such as OX40 and 4-1BB (CD137) [6, 7]. In addition to enhancing the activation of effector T (Teff) cells, it may also be useful to inhibit regulatory T (Treg) cells or other immuno-suppressive cell populations within tumors [8]. Indeed, Treg modulation may contribute towards the efficacy of certain cancer therapies. For example, recent mechanistic studies indicate the anti-CCR4 mAb mogamulizumab, approved for the treatment of relapsed adult T cell leukemia (ATL) in Japan [9], could reduce the number of Treg cells in cancer patients [10]. Meta-analysis of clinical data indicates that increased CD8^+^ Teff/Treg ratios correlate to improved prognosis in multiple cancer types [11]. Identifying novel drug targets expressed by Teff and/or Treg cells will allow for novel drug combinations, and ultimately may lead to enhanced clinical response rates and bring significant benefit to patient populations that currently fail to respond to existing therapies [12].

The search for a unique cell-surface marker that reproducibly identifies human or mouse Treg populations has been challenging. As such, Tregs are currently categorised by the expression of two cell surface markers, CD4 and CD25 [13], and the intracellular transcription factor Foxp3 [14]. Previous attempts have been made to identify novel Treg surface markers using unbiased antibody screening of hybridomas from mice immunised with human Treg cells [15]. However, despite extensive screening, the antibodies isolated from this Treg immunisation approach were only able to identify existing, known markers such as CD25 and MHC class II, which on their own have limited Treg selectivity.

Our previous work has shown that phage display enrichment of diverse antibody libraries by panning on cells, followed by antibody screening for selective binding or desired function can be an effective, target-agnostic approach to identify novel markers and indeed therapeutic targets [16–18]. We have also demonstrated that phage display may have advantages over hybridoma-based screening, due to the ability to rationally deselect antibodies to known or abundant antigens, therefore enabling access to wider pools of targets during the screening process [17, 18].

For these reasons, we employed a target-agnostic phage display screening approach on human Treg cells to find antibody mimetics with preferential binding to Treg, rather than Teff, cells. For optimal performance during the phage display step we used designed ankyrin repeat proteins (DARPins) [19–22], antibody mimetics which are particularly suitable for cell-based selections, due to high levels of display on phage [18]. To our surprise, all of the most selective DARPins prioritised from this screen were found to bind specifically to tumor necrosis factor receptor 2 (TNFR2, TNFRSF1B), a receptor for membrane-bound TNF-α [23]. Some of the DARPins were found to also agonise TNFR2, activating the NF-κB signalling pathway in primary human Treg cells and Jurkat T cells transfected with TNFR2. Furthermore, we demonstrated, using mouse-reactive TNFR2-specific agonists, that we could enhance anti-tumor immunity in the CT26 syngeneic tumor model. Together these data provide support for TNFR2-specific agonists as potential cancer immunotherapy drugs.

## RESULTS

### Target-agnostic screening for human Treg cell selectivity

**Figure 1:**
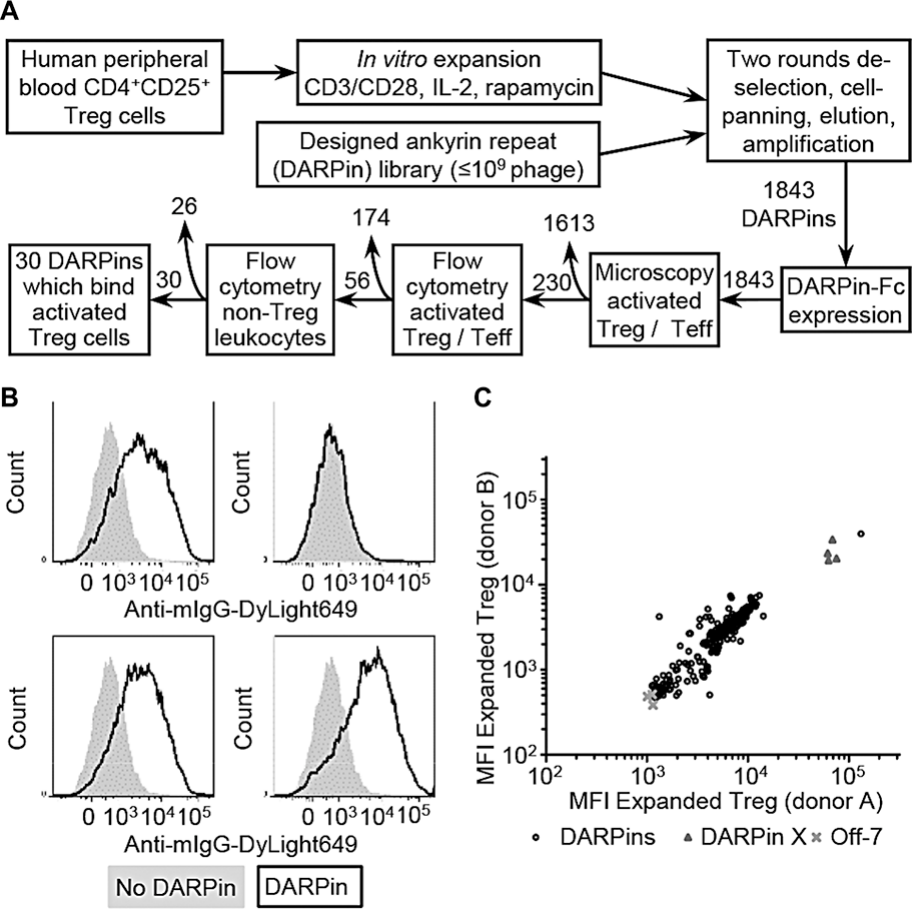
Isolation of designed ankyrin repeat proteins (DARPins) which bind human Treg cells (**A**) CD4+CD25+ Treg cells isolated from healthy donor peripheral blood mononuclear cells (PBMCs) were activated, expanded *in vitro* for fourteen days, and used for cell-binding affinity selections with a diverse library of DARPins. Output DARPins were screened for binding Treg, CD4+ Teff cells, and other leukocyte populations by high-throughput microscopy and flow cytometry, resulting in the isolation of thirty DARPins with preferential binding for human Treg cells. (**B**) Example data showing binding of four distinct DARPin-Fc molecules to activated Treg cells. (**C**) Median fluorescence intensity (MFI) values for DARPins binding to expanded Treg cells from two independent donors. DARPin X is a positive control which binds to all T cells; Off-7 is a negative control.

To identify Treg cell-surface proteins by phage display affinity selections, highly pure populations of human CD4^+^CD25^+^ Treg cells were isolated from healthy donor peripheral blood as described in *Materials and Methods* (Figure 1A) [24]. Purity was assessed by staining for Foxp3 (Supplementary Figure S1A) and Treg phenotype confirmed using *in vitro* suppression assays (Supplementary Figure S1B). Phage display cell affinity selections were performed using a DARPin library, de-selecting using a panel of recombinant T cell markers (listed in *Materials and Methods*). The selection output following two rounds of selections was sub-cloned, and sequencing identified 1843 unique DARPin clones from a total population of 2816 clones sequenced (65% sequence diversity). The binding of the 1843 unique potential Treg binding DARPins, expressed as mouse IgG_2a_ Fc fusion proteins, to activated human Treg and CD4^+^ Teff cells was investigated using high-throughput microscopy and flow cytometry (Figure 1A–1C). A group of 56 DARPins were identified that bound to Treg and not to activated CD4^+^CD25^−^ Teff cells; from these a group of 26 DARPins were deprioritised due to their binding activated human NK cells, B cells, monocytes, or broad populations of cells amongst human peripheral blood leukocytes. Finally, thirty DARPins were identified (hereafter referred to as TREG001 to TREG030), which bound selectively to activated human Treg cells.

### DARPins bind to TNFR2

**Figure 2:**
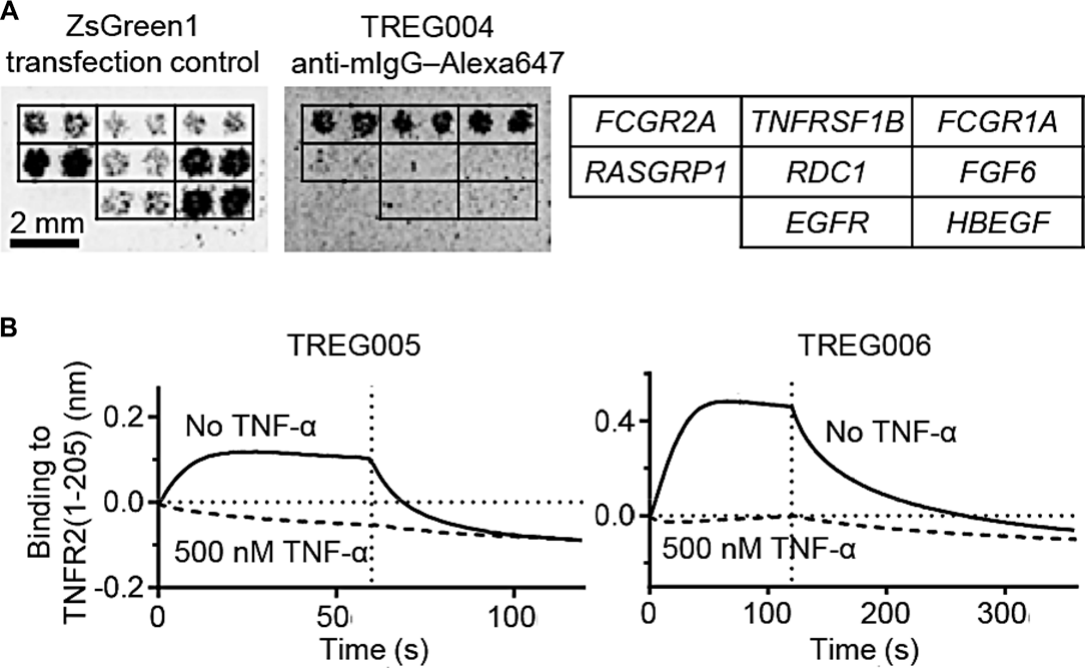
Treg-binding DARPins bind to TNFR2 in a TNF-α-competitive manner (**A**) Binding of TREG DARPins to HEK293 cells expressing *TNFRSF1B*, FcγRs (positive controls) or other genes (negative controls) was investigated using membrane protein expression arrays. (**B**) Bio-layer interferometry was used to investigate the interaction of immobilised TREG005 and TREG006 with 100 nM soluble TNFR2(1-205) (solid lines) or 100 nM TNFR2(1-205) pre-incubated with 500 nM human TNF-α (dashed lines). Vertical dotted lines indicate transition from association to dissociation phases.

To investigate epitope redundancy amongst the thirty Treg-binding DARPins, TREG001 and TREG002 were arbitrarily chosen and each was labelled with biotin and used to stain Treg cells following pre-incubation with unlabelled samples of each of the thirty DARPins of interest (Supplementary Figure S2). In every case, pre-incubation reduced the extent of biotinylated TREG001 and TREG002 binding to Treg cells, indicating that the thirty DARPins bound to the same antigen. To identify this antigen, TREG001, TREG002, and six others were tested for binding to a membrane protein expression library array. The DARPins were observed to bind to cells expressing *TNFRSF1B*, which encodes TNFR2. This result was then confirmed using a limited panel of genes including *TNFRSF1B*, genes encoding Fcγ receptors, and a protein for which non-specific binding is frequently observed (*RASGRP1*; Figure 2A).

To further confirm TNFR2 was the target antigen for the Treg DARPins, activated Treg cells were incubated with goat anti-human TNFR2 polyclonal antibodies (pAb), which were observed to result in loss of binding by DARPins (Supplementary Figure S3A). DARPins TREG005 and TREG006 were then chosen for more detailed analysis, on the basis of high staining intensity, high yields following transient transfections, and low levels of aggregation in solution (data not shown). TREG005 and TREG006 bound to recombinant TNFR2 but not to TNFR1, TNFR3, or osteoprotegerin, which has high sequence similarity to TNFR2 (Supplementary Figure S3B).

### Characterising the interaction between DARPins and TNFR2

TREG005 and TREG006 were observed to bind to Treg cells with half-maximal binding at concentrations of ∼100 pM and 10 pM respectively (EC_50_ values; Supplementary Figure S3C) and to constructs incorporating the third and fourth extracellular cysteine-rich domains of TNFR2, but not to a TNFR2 construct lacking these domains (Supplementary Figure S4A, S4B). Binding of TNFR2 to immobilised TREG005 and TREG006 was abrogated by pre-incubation of TNFR2 with TNF-α (Figure 2B), and binding to immobilised TNF-α was reduced by pre-incubation with TREG005 and TREG006 but not control DARPin E3_5 (Supplementary Figure S5). Therefore these DARPins bound to TNFR2 in a TNF-α-competitive manner.

### TREG005 and TREG006 induce NF-κB signalling in Treg cells

**Figure 3:**
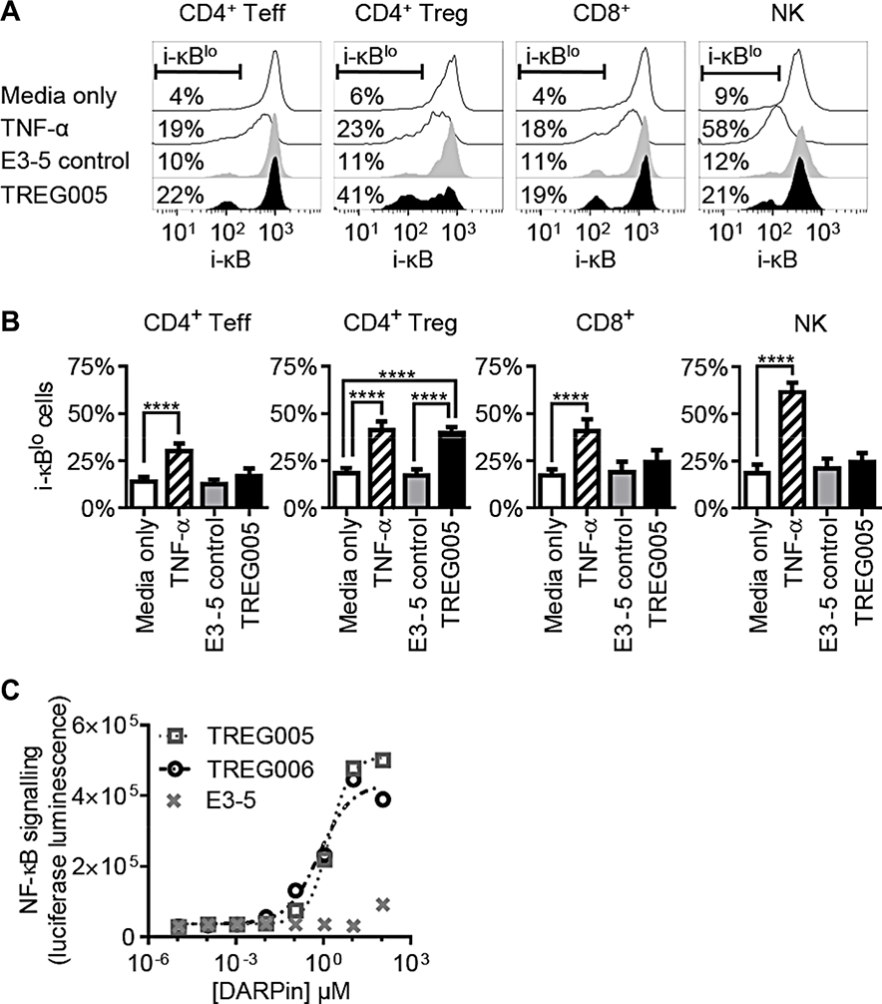
TNFR2-binding DARPins induce NF-κB signalling in Treg cells (**A**) Human PBMCs were incubated with 10 μg/ml TREG005, control DARPin E3-5 or 10 ng/ml TNF-α for 15 min, stained for lymphocyte sub-set markers and intracellular inhibitor of κBα (i-κBα). (**B**) Summary of i-κB degradation data for multiple donors (*n* = 10, error bars indicate SEM; significance assessed using 2-way ANOVA). (**C**) Jurkat E6.1 cells transfected to express TNFR2 and NF-κB-responsive luciferase were incubated with DARPins for 5.5 hrs, after which luciferase expression was assessed by luminescence (representative of three independent repeats).

**Figure 4:**
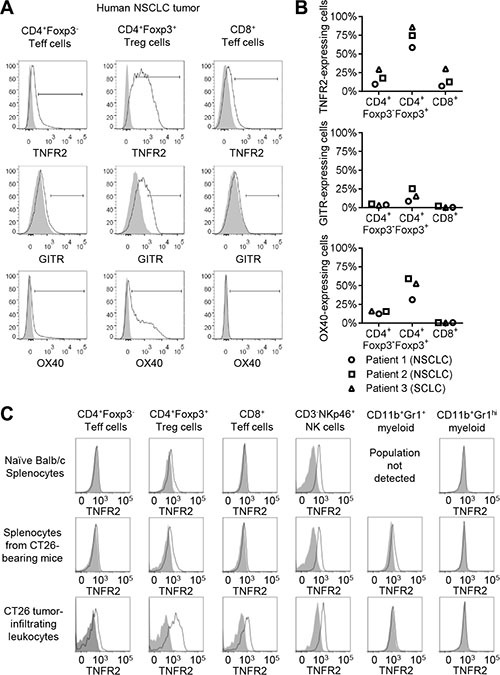
TNFR2 expression within tumors (**A**) Tumor samples from three lung cancer patients were analysed for expression of TNFR2, glucocorticoid-induced TNF-related protein (GITR), OX40 and T cell lineage markers by flow cytometry. Data shown are for Patient 2 in panel (**B**). (B) Summary of TNFR2, GITR and OX40 expression for tumor-infiltrating T cells from three lung cancer patients. (**C**) Spleens and tumors from Balb/c mice implanted sub-cutaneously with CT26 tumor cells or spleens from untreated animals were analysed for expression of TNFR2 and lineage markers by flow cytometry (representative of eight tumor-bearing animals and three non-tumor-bearing animals in three independent experiments).

TNFR2 signalling promotes T cell activation by activating the NF-κB signalling pathway via degradation of inhibitor of κB (i-κB) proteins [25]. To investigate the potential effects of TNFR2-binding DARPins on T cells, human PBMCs were cultured with negative control DARPin E3_5 or TREG005 for 15 min, followed by analysis of i-κBα degradation by flow cytometry. Lymphocyte sub-sets were identified using mAbs specific for TCRαβ, CD4, CD8, CD25, CD56, and CD127. TNF-α was used as a positive control to induce i-κBα degradation in all cells via TNFR1 (Figure 3A, 3B). TREG005 induced degradation of i-κBα within CD4^+^CD25^+^CD127^lo^ Treg cells, while effects on non-Treg cell populations were less pronounced and were not significant (Figures 3A, 4B). To a lesser extent, TREG006 also induced degradation of i-κBα within Treg cells (data not shown). Degradation of i-κBα in Treg cells in response to TNFR2-binding DARPins was consistent with expression of TNFR2 by Treg cells (Supplementary Figure S6A). Furthermore, using Jurkat-based reporter cells, TREG005 and TREG006 were observed to induce NF-κB-dependent expression of firefly luciferase (Figure 3C). This activity depended on transfection to express TNFR2, and was enhanced by cross-linking with a polyclonal anti-human Fcγ antibody (data not shown), indicating TREG005 and TREG006 are TNF-α-independent agonists which induce NF-κB signalling by clustering TNFR2 molecules. A more detailed investigation into the downstream effects of DARPin-mediated TNFR2 signalling is on-going and will be reported elsewhere.

### Profiling TNFR2 expression

TNFR2 expression has been widely reported for Treg cells and other T cell populations [26–28]. To profile TNFR2 expression, human PBMCs were cultured in the presence or absence of PHA-P and IL-2, and then stained for binding by anti-TNFR2 or control mAbs and a lineage panel comprising CD3, CD4, CD8, CD25, CD56 and Foxp3. TNFR2 was expressed by unstimulated CD4^+^Foxp3^+^ Treg cells, but not by other evaluated unstimulated lymphocyte populations (Supplementary Figure S6A). Following PHA-P/IL-2 stimulation, TNFR2 was additionally expressed by CD4^+^Foxp3^−^ and CD8^+^ Teff cells, and NK cells. Next, PBMCs from HLA-A^+^ ndividuals with pre-determined reactivity to cytomegalovirus (CMV) pp65 antigen were incubated with pp65 peptide NLVPMVATV and profiled for TNFR2 expression. In addition to TNFR2 expression by Treg cells, greater intensity expression was observed for pp65-specific CD8^+^ T cells (Supplementary Figure S6B, S6C). Of note, TNFR2 expression was observed for all or most pp65-specific CD8^+^ T cells (Supplementary Figure S6C, S6D). These data indicate that TNFR2 is expressed by unstimulated Treg cells, and is also expressed by activated Teff cells and NK cells.

Next, TNFR2 expression by tumor-infiltrating T cells was investigated. Expression of GITR and OX40 by tumor-infiltrating T cells was also investigated because, like TNFR2, these are co-stimulatory TNFRSF members which have been reported to be expressed by Treg cells [29]. Tumor samples from three lung cancer patients were analysed by flow cytometry, staining for CD19, CD3, CD4, CD8, Foxp3, TNFR2, GITR and OX40 (Figure 4A, 4B). High levels of TNFR2 expression were detected for CD4^+^Foxp3^+^ regulatory T cells, while lower levels were detected for CD4^+^Foxp3^−^ and CD8^+^ T cells (Figure 4A, 4B). Similarly, the highest levels of GITR and OX40 were also detected for CD4^+^Foxp3^+^ Treg cells and lower levels for CD4^+^Foxp3^−^ Teff cells. In contrast to TNFR2, very low or undetectable levels of GITR and OX40 were observed for CD8^+^ T cells. Together, these data indicate that TNFR2 is expressed by Treg and Teff cells within lung tumors; TNFR2 has a similar expression profile to OX40 and GITR, and is additionally expressed by tumoral CD8^+^ T cells.

To investigate TNFR2 expression within a broader sample of human cancers, publicly available gene expression data were analysed (The Cancer Genome Atlas). Expression of *TNFRSF1B* was observed to correlate with *CD3E* expression (a marker for T cell infiltration of tumors) [30] for multiple tumor types, including bladder urothelial carcinoma, breast invasive carcinoma, head and neck squamous cell carcinoma, kidney carcinoma, lung squamous cell carcinoma, prostate adenocarcinoma, melanoma, and uterine corpus endometrial carcinoma (Supplementary Figure S7). These data support the hypothesis that tumor-infiltrating Treg and other T cells in multiple tumor types express TNFR2 [31, 32].

Next, to profile TNFR2 expression within a pre-clinical tumor model, immuno-competent mice were implanted with CT26 colorectal tumor cells. CT26 tumors may provide a good model for immune responses to tumors which are responsive to cancer immunotherapy, due to a high mutational burden [33]. TNFR2 expression was observed for CD4^+^Foxp3^+^ Treg and NKp46^+^ NK cells in all tissues examined, and was additionally observed for tumor-infiltrating CD4^+^Foxp3^−^ and CD8^+^ T cells, and for splenic CD11b^+^Gr1^+^ myeloid cells from tumor-bearing animals (Figure 4). Tumor-infiltrating Treg cells expressed the highest levels of TNFR2. Mouse NK cell TNFR2 expression was constant for all tissues investigated, and likely reflects a species difference between mice and humans. Together, these data indicate that TNFR2 is expressed by Treg cells and by effector T cells in the context of anti-tumor immune responses.

### TNFR2 mAbs enhance anti-tumor immunity in immuno-competent mice

The TNFR2 agonists identified by phenotypic screening did not cross-react with mouse TNFR2 since they were raised against human Treg cells. Therefore mouse-reactive TNFR2 agonist mAbs were sourced and used as surrogates to explore anti-tumor immunity in immuno-competent mice. Clone TR75-54.7 hamster anti-mouse TNFR2 mAb was previously found to compete with TNF-α, and to act as a TNFR2 agonist when cross-linked *in vitro*, detected by proliferation of the CT6 T cell-line [34]. Here, TR75-54.7 was confirmed to be a TNF-α-competitive TNFR2 agonist using bio-layer interferometry and a cell-based NF-κB reporter system (Supplementary Figure S8 and S9), therefore indicating this mAb is a suitable surrogate for the TNFR2-binding agonist DARPins. An additional anti-TNFR2 mAb (clone TR75-89) was investigated, which is also a TNFR2 agonist but does not compete with TNF-α [Supplementary Figures S8 and S9, and 34]. There is now broad evidence that mAbs targeting TNFRSF members can act as agonists *in vivo* due to cross-linking by FcγR-expressing cells [35, 36]. Binding to recombinant mouse FcγRII and FcγRIII was observed for anti-TNFR2 mAbs TR75-54.7 and TR75-89, although no interaction with FcγRI or FcγRIV was observed (Supplementary Table S1), indicating these mAbs can be cross-linked by a sub-set of mouse FcγRs but should not be expected to mediate antibody-dependent cellular phagocytosis (ADCP).

**Figure 5:**
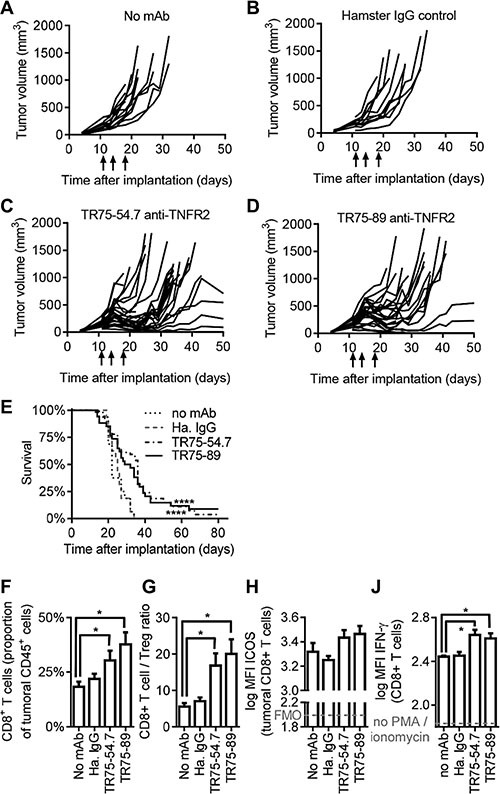
Anti-TNFR2 mAbs inhibit tumor growth in mice Balb/c mice were implanted with CT26 cells and following 11, 14 and 18 days of tumor growth were injected i.p. with either (**A**) saline, (**B**) 100 μg hamster IgG control mAb, (**C**) 100 μg TNF-α competitor anti-TNFR2 mAb (clone TR75-54.7) or (**D**) 100 μg TNF-α non-competitor anti-TNFR2 mAb (clone TR75-89; arrows on graphs indicate dosing). Data for (A, B) sixteen animals per group and (C, D) 34 animals per group in two independent studies. (**E**) Survival of animals as determined by time to reach a humane end-point based on tumor size (*p* < 0.0001 by log-rank test). (**F**–**J**) Animals were administered anti-TNFR2 or control mAbs on days 11 and 14 after implantation with CT26, sacrificed on day 18, and intra-tumoral lymphocytes analysed by flow cytometry. (F, G) Intra-tumoral CD8+ T cells and CD4+Foxp3+ Treg cells were enumerated as a proportion of tumoral CD45+ cells (mean +/− SEM, *n* = 12, *p* < 0.05 by 2-way ANOVA). (H) ICOS expression by CD8+ T cells (mean +/– SEM, *n* = 6). FMO indicates background staining using isotype-matched irrelevant control mAbs. (J) 18 days after tumor implantation, splenocytes were stimulated *ex vivo* with PMA/ionomycin in the presence of brefeldin-A for 5.5 hrs to assess cytokine synthesis by flow cytometry (mean +/– SEM, *n* = 6, *p* < 0.05 by 2-way ANOVA)

Growth of CT26 tumors in immuno-competent mice was inhibited by administration of TNFR2 mAbs, compared to control animals which received saline or hamster IgG control mAbs (Figure 5A–5D). Median survival (median time taken to reach a consistent humane end-point based on tumor size) was 36 and 30.5 days after implantation for animals which received TR75-54.7 or TR75-89 anti-TNFR2 mAbs, compared to 22 days or 25 days for animals which received saline or hamster IgG control mAb respectively (*p* < 0.0001; Figure 5E). Based on reported serum half-lives of approximately two days [34], approximately 90% of the total exposure to anti-TNFR2 mAbs occurred within ten days following the first dose. Therefore, the duration of tumor growth inhibition and enhanced survival were similar to the exposure to TNFR2 agonists.

Complete tumor regression was observed for two out of 34 animals which received anti-TNFR2 mAb TR75-54.7, and three out of 34 animals which received TR75-89 (Figure 5E). No tumor growth was observed when these animals were re-challenged with CT26 cells at least thirty days after tumor regression, while CT26 cells implanted into previously untreated control animals grew normally (data not shown). This indicates that TNFR2 mAbs induced long-lasting immunological memory against CT26 tumor cells.

To investigate the mechanism by which TNFR2-binding mAbs enhanced anti-tumor immunity, separate groups of CT26 tumor-bearing animals which received TNFR2 mAbs were sacrificed eighteen days after implantation and analysed by flow cytometry. Tumoral CD8^+^ T cell populations were greater for animals that received anti-TNFR2 mAbs than for controls (as a proportion of total tumoral CD45^+^ cells, Figure 5F), resulting in increased CD8^+^ T cell/Treg ratios (Figure 5G). No statistically significant effects on CD4^+^Foxp3^−^ Teff cells, CD4^+^Foxp3^+^ Treg cells, CD3^−^NKp46^+^ NK cells or CD11b^+^Gr1^+^ myeloid cells were observed, and no effects were observed for any of these populations within spleens or tumor-draining inguinal lymph nodes (data not shown). Intratumoral CD8^+^ T cells expressed slightly higher levels of inducible T cell costimulator (ICOS) in response to TNFR2 agonist mAbs (Figure 5H), and splenic CD8^+^ T cells from mice which received TNFR2 agonist mAbs expressed higher levels of IFN-γ than cells from control animals, when stimulated with PMA/ionomycin in the presence of Brefeldin-A (Figure 5J).

## DISCUSSION

Phenotypic screening for antibody mimetics with selectivity for human Treg cells has highlighted TNFR2 as a potential therapeutic target for cancer immunotherapy. This approach was made possible by high purity isolation and *in vitro* expansion of human peripheral blood Treg cells, and through the use of DARPins, antibody mimetics which exhibit enhanced levels of display on phage [18–22]. Rapid target identification was achieved using membrane protein expression arrays (Figure 2A). Some of the TNFR2-specific DARPins were observed to induce i-κBα degradation in resting human Treg cells (Figure 3B), and to mediate NF-κB signalling in Jurkat T cells expressing TNFR2 (Figure 3C). The NF-κB signalling pathway is a key determinant of T cell activation [25], and has been implicated in down-regulation of Treg-mediated suppressive activity [37]. These observations indicate the potential for TNFR2-specific agonists to therapeutically enhance T cell-mediated immunity.

TNFR2 expression by melanoma, colorectal cancer and lung cancer TILs has been reported previously [31], but data for expression by specific T cell sub-sets were not available. Here, TNFR2 expression was observed for Treg cells within human lung tumor samples and healthy donor PBMCs (Figure 4A, 4B, Supplementary Figure S6A). Additionally, lower intensity TNFR2 expression was observed for tumor-infiltrating CD4^+^Foxp3^−^ and CD8^+^ Teff cells, and for PBMC-derived Teff cells following *in vitro* stimulation with PHA-P/IL-2 or specific cognate antigenic peptide (Supplementary Figure S6). TNFR2-binding DARPins were not initially observed to bind to CD3/CD28-stimulated CD4^+^ Teff in the cell-based screening cascade (Figure 1), due to minimal TNFR2 expression by these cells. This may reflect a difference in the strength of T cell stimulation provided by anti-CD3/CD28 mAbs compared to other polyclonal stimuli or cognate antigenic stimulation. Together, these data support the idea that TNFR2 agonists may provide a means to therapeutically modulate tumoral Treg and Teff cells.

Therapeutic modulation of TNFR2 was investigated in mice bearing CT26 sub-cutaneous syngeneic tumors. Within these tumors, high levels of TNFR2 were found to be expressed by CD4^+^Foxp3^+^ Treg cells, with lower levels for CD4^+^Foxp3^−^ and CD8^+^ T cells (Figure 4C). That is, a similar expression pattern was observed as for T cells within human NSCLC tumors. TNFR2 expression was also observed for splenic CD4^+^Foxp3^+^ Treg cells in naïve and tumor-bearing animals, and human peripheral blood Treg cells, confirming constitutive expression by Treg cells in the periphery of both species. These data demonstrate the relevance of the CT26 syngeneic tumor model for investigating TNFR2 agonists in cancer immunotherapy.

High-specificity binding to targets is a key advantage of biologics over small molecule drugs, but frequently also leads to a lack of cross-reactivity with non-primate species. Hence, research within the field of cancer immunotherapy is frequently based on rodent-reactive surrogate reagents. For example, mouse-reactive anti-CTLA-4 and anti-PD-L1 mAbs were used pre-clinically prior to clinical evaluation of human-reactive mAbs targeting these pathways [38, 39, and reviewed in 40]. Accordingly, a mouse-reactive TNF-α-competitive TNFR2 agonist mAb was used here as a surrogate for the TNFR2 agonist DARPins. This TNFR2 agonist mAb enhanced tumor infiltration and IFN-γ synthesis by CD8^+^ T cells in the CT26 tumor model, resulting in tumor growth inhibition and prolonging survival by approximately 5–15 days (Figure 5). These data provide *in vivo* support for exploring TNFR2 agonists in cancer therapy.

Antibodies and related molecules can enhance the immune response to cancer either through antagonism of immunosuppressive molecules, such as anti-CTLA-4 or PD-1 mAbs, through agonist activity, as previously described for mAbs targeting TNFRSF family members including GITR, OX40, CD40, and 4-1BB [41], or by depleting specific cell-types [42]. In principal, the TNF-α-competitive anti-TNFR2 mAb used here could influence anti-tumor immune responses through blockade of TNF-α binding to TNFR2. However, very similar effects on tumor growth were observed for a different TNFR2 agonist mAb which does not compete with TNF-α, demonstrating that antagonism did not contribute appreciably to the therapeutic activity of the anti-TNFR2 mAbs. Similarly, while intra-tumoral depletion of Treg cells was recently found to contribute to the anti-tumor activity of CTLA-4 mAbs in mouse models [42], intra-tumoral and splenic Treg populations were not significantly depleted by TNFR2 mAbs, and the hamster anti-TNFR2 mAbs used here did not bind detectably to mouse FcγRI or FcγRIV, indicating these mAbs are unlikely to induce ADCP (Supplementary Table S1). The ability of the mouse-reactive TNFR2 mAbs to induce TNFR2 signalling has been described previously [34], was confirmed here using *in vitro* reporter assays (Supplementary Figure S9), and is supported by the observation that these mAbs bind to, hence may be cross-linked by, mouse FcγRII and FcγRIII (Supplementary Table S1). In summary, the most likely mechanism for TNFR2 mAb-induced tumor growth inhibition is TNFR2 agonism, and associated changes in T cell function.

In contrast to the near-ubiquitous expression of TNFR1, expression of TNFR2 is confined to specific populations of cells including regulatory T cells, activated effector T cells and NK cells. TNFR2 agonist mAbs were observed to increase the numbers of CD8^+^ T within tumors, resulting in increased CD8^+^ Teff/Treg ratios, to increase the expression of ICOS by tumoral CD8^+^ T cells, and to enhance the level of IFN-γ synthesis by CD8^+^ T cells following *ex vivo* stimulation (Figure 5). Although mouse NK cells were observed to express TNFR2 (Figure 4), likely reflecting a species-specific difference between mice and humans, no effects on NK cell frequency or expression of activation markers were observed *in vivo* in response to TNFR2 mAbs (Figure 5). This suggests that TNFR2 molecules expressed by NK cells do not contribute towards the therapeutic mechanism of action for TNFR2 agonists in this model. Taken together, the pharmacodynamic data indicate a central role for CD8^+^ T cells in the response to TNFR2 agonists, and are also compatible with TNFR2-mediated down-regulation of Treg cell suppressive activity [37, 43].

TNFR2 signalling has previously been shown to enhance T cell activation and decrease Treg-mediated suppression [43–45], possibly via phosphorylation of the transcription factor Foxp3 [46], and via activation of the NF-κB signalling pathway in Treg cells [37]. Proliferation of adoptively transferred Ag-specific T cells in an LCMV glycoprotein-driven tumor model was impaired for TNFR2-deficient T cells [47], supporting the idea that TNFR2 contributes to anti-tumor immunity. However, other reports have suggested that TNFR2 agonism could be detrimental towards anti-tumor immunity, either by inducing activation-induced cell death (AICD) of T cells [48, 49], or by enhancing the activation and suppressive activity of Treg cells [50] or myeloid-derived suppressor cells [51]. Inhibition of TNFR2 using anti-sense oligonucleotide technology was observed to reduce experimental liver metastasis by H-59 cells in mice [52], and in one report, growth of syngeneic tumors in TNFR2-deficient mice was reduced compared to wild-type animals [52], while in another study, tumor growth in TNFR2-deficient animals was initially increased but at later time-points was reduced compared to wild-type animals [49]. While we cannot rule out any detrimental effects of TNFR2 agonism on anti-tumor immunity, overall, the net effect of administering TNFR2 agonist mAbs to tumor-bearing mice was clearly to enhance anti-tumor immunity.

Clinical evaluation of TNFR2-specific agonists could be performed using biologics based on DARPins described here, supported by previous investigational administration of DARPins to patients [53]. Alternatively, human-reactive TNFR2 agonist mAbs could be generated by phage display or immunisation approaches. We anticipate TNFR2 agonists will be most effective for treating tumors with a T cell-inflamed phenotype [54]. Publicly available data indicate a positive correlation between *CD3E* and *TNFRSF1B* expression (Supplementary Figure S7), suggesting that for many T cell-inflamed tumors, target expression may be sufficiently high to support responses to TNFR2-targeted therapy. In these settings, dominant immunological tolerance is frequently maintained within the tumor micro-environment by the PD-1/PD-L1 axis. Consequently, novel immuno-oncology drugs are very often investigated in combination with PD-1 or PD-L1 blockade [12]; this is an attractive combination strategy for TNFR2 agonists, which should be tested pre-clinically prior to clinical investigation. On the basis of the favourable pre-clinical activity observed here for TNFR2 agonists, we propose that TNFR2-specific agonist biologics represent an attractive candidate for development as cancer immunotherapy drugs.

## MATERIALS AND METHODS

### Isolation and activation of human CD4^+^ Treg and Teff cells

Healthy donor leukapheresis samples were obtained from Research Blood Components (Brighton, MA) or the UK National Health Service Blood and Transfusion services (NHSBT, Addenbrooke's Hospital, Cambridge). Peripheral blood mononuclear cells (PBMCs) were isolated by Ficoll-Paque centrifugation (GE Healthcare). CD4^+^CD25^−^ Teff and CD4^+^CD25^+^ Treg cells were isolated using negative selection followed by CD25 selection (Dynal CD4^+^CD25^+^ Treg Kit, Invitrogen; or human CD4^+^ T cell enrichment and human CD25 positive selection kits, StemCell Technologies). CD4^+^CD25^+^ cells were cultured in X-vivo15 medium (Lonza) supplemented with 5% human AB serum (Life Technologies), HEPES (Life Technologies), 1000 U/mL interleukin (IL)-2 (Roche Diagnostics) and 100 nM rapamycin (Sigma-Aldrich). Anti-CD3/CD28 beads were added (Human Treg expander kit, Life Technologies) at a cell:bead ratio of 1:4. Cells were cultured in a humidified incubator at 37°C/5% CO_2_ for 7–9 days, with addition of fresh medium containing 500 U/mL IL-2 every 2–3 days. CD3/CD28 beads were then removed, the cells washed and fresh medium containing 100 U/mL of IL-2 and 100 nM rapamycin was added every 2–3 days. To assess purity, cells were fixed and permeabilised (Foxp3/Transcription Factor Staining Buffer Set, eBioscience), stained using PE-conjugated anti-Foxp3 mAbs (clone PCH101) and analysed by flow cytometry (FACSCanto II, BD Biosciences). CD4^+^ effector T cells were activated using anti-CD3/CD28 beads (Human T-activator or Treg expander beads, Invitrogen) and 30 U/mL IL-2 (Roche Diagnostics) for four days.

### Cell-based DARPin phage display affinity selections

DARPins have been described previously [19, 21]. Two rounds of cell-based affinity selections were each performed on 10^7^ activated expanded Treg cells originating from seven donors using a DARPin phage display library containing up to 10^9^ binding members, as described previously [17, 18]. The following recombinant human proteins were included as de-selection antigens: TCR α and β chains, CD5 (Cambridge Biosciences); CD69, CD3ε, and ITGB2 (Sino Biological); CD2 (Abcam); CD132, CD122, CD39, IL-10Rα, sCD4, CD109, IL-1R, IL-6Rα, IL-14Rα, IFN-γR1, CD45, CD38, TNFRI, 4-1BB, CD30, GITR, PD-1, B7-H1, CD44, CD25, CD27, and CD28 (R&DSystems). DARPins isolated from two rounds of selections were reformatted as either human IgG_1_ Fc domain fusions or mouse IgG_2a_ Fc domain fusions and expressed in HEK293 cells (ATCC).

### Staining immune cells with DARPins

Mouse IgG_2a_ DARPin-Fc fusions were initially screened for binding Treg and Teff cells using 8200 Cellular Detection System (Applied Biosystems) and flow cytometry experiments (Figure 1 only). For subsequent experiments, purified human IgG_1_ Fc-tagged DARPins were used at 5 μg/mL. Where appropriate, cells were pre-incubated with 200 μg/mL goat anti-TNFR2 pAb (R&DSystems). Cells were incubated with DARPins for 90 min at 4°C (or in the case of titrations, on ice with agitation for 16 h) washed, incubated with DyLight649-labelled goat anti-mouse or anti-human IgG pAb (Jackson Immunoresearch) for 45 min at 4°C and washed again. Alternatively, cells were incubated with unlabelled DARPins at a concentration of 400 μg/mL for 90 min, after which an equal volume containing biotinylated DARPins at 50 μg/mL was added without washing, the cells incubated for a further 45 min, washed, stained using APC-labelled streptavidin (eBioscience) for 45 min and washed again. Cells were fixed and analysed by flow cytometry (FACSCantoII, BD Biosciences).

### Membrane protein expression arrays

Mouse IgG_2a_ Fc-tagged DARPins were screened for binding to 2505 human plasma membrane proteins expressed in HEK293 cells using cell microarray technology [Retrogenix, 54]. Transfection efficiency was assessed by simultaneous transfection with pIRES-hEGFR-IRES-ZsGreen1. An AlexaFluor647-labelled anti-mouse IgG detection antibody (Life Technologies) was used to detect DARPin binding. To confirm binding, primary hits were re-expressed in duplicate and probed with DARPins or a negative control mouse Fc-fusion protein.

### ELISAs

Recombinant TNFR1, TNFR2 (R&D Systems), TNFR3 (LTBR, Stratech) or Osteoprotegerin (TNFRSF11B, Stratech) or recombinant FLAG-His_10_ tagged TNFR2 ECD constructs, described in detail in *Supplementary Materials and Methods*, were immobilised on Maxisorb 96-well plates (Nunc). Plates were washed with PBS, blocked using 3% non-fat milk in PBS, then washed again. DARPin-Fcs or control antibodies at 2 μg/mL in blocking solution were incubated on the plates for 1 h at RT. Anti-TNFR2 mIgG_2a_ clone 22235 (R&D Systems), anti-His mIgG_2b_ clone His.H8 (Millipore) and mIgG_2b_ isotype control mAbs were used as controls. Plates were washed with PBS-Tween, then incubated with peroxidase-conjugated goat anti-human or anti-mouse IgG pAb (Sigma) in 3% non-fat milk/PBS-Tween. Plates were washed again with PBS-Tween and developed using 3,3′,5,5′ Tetramethylbenzidine (TMB) liquid substrate, followed by 0.5 M H_2_SO_4_, and absorption at 450 nm determined (Envision, Perkin Elmer).

### TNF-α competition binding analyses

Recombinant human TNF-α (R&DSystems) or anti-mouse TNFR2 mAbs TR75-54.7 and TR75-89 (R&DSystems) were biotinylated using EZ-Link Sulfo-NHS-LC-biotin (Pierce) then buffer exchanged into PBS (Sigma) using a Zeba-Spin 7k MWCO 0.5 mL column (Pierce). Competition experiments were performed by Biolayer Interferometry (OctetRed384, ForteBio). In each case, one protein component (i.e. DARPin-Fc, biotinylated TNF-α or biotinylated anti-mouse TNFR2 mAb) was immobilised on either streptavidin- or protein A-coated Dip and Read™ biosensors (ForteBio) as appropriate (load step, typically 120 s), then the response was measured while the sensors were sequentially transferred to wells containing buffer only (baseline step, 60 s), ligand proteins ± excess competitor proteins (association step, typically 180 or 300 s), then buffer only (dissociation step, varying durations). Assays were performed at 25°C with 1000 rpm shaking, and using proteins diluted in PBS containing 0.01% BSA 0.005% Tween-20.

### i-κBα degradation assay

PBMCs were isolated from fresh whole blood donated by volunteers of the Cambridge BioResource with the approval of the Cambridge South Research Ethics Committee (UK). PBMCs were cryopreserved, defrosted, and cultured overnight in RPMI1640-based medium at 37°C/5% CO_2_. Cells were washed and incubated at 10^6^ cells per mL in 500 μL medium per well, supplemented where appropriate with 10 ng/mL TNF-α or 10 μg/mL DARPins for 15 min at 37°C/5% CO_2_. Cells were re-suspended, washed once with medium, and stained in 100 μL per sample medium supplemented with membrane staining antibodies (TCRαβ-FITC clone IP26, CD4-PerCP-Cy5.5 clone Okt4, CD8-BV711 clone SK1, CD56-PE-Cy7 clone HCD56, CD25-APC clone M-A251, CD127-BV421 clone eBioRDR5) for 15 min at room temperature, protected from light. Fixation/permeabilisation was performed according to manufacturer's instructions (Introprep, Beckman Coulter). 2 μL per sample of PE-labelled anti-ikBα (clone L35A5, Cell signalling) or isotype control mAbs was added, and the cells incubated for 15 minutes at RT, protected from light. Cells were washed, fixed and analysed promptly by flow cytometry (Fortessa, BD Biosciences).

### TNFR2 signalling assays

Jurkat E6.1 cells were transfected to express TNFR2 and NF-κB-dependent luciferase (described in *Supplementary Materials and Methods*). 10^5^ cells per well were added to opaque 96-well tissue culture plates (Corning) in 200 μL per well of RPMI-based medium supplemented with DARPins or mAbs, incubated at 37°C/5% CO_2_ in a humidified incubator for 5.5 h, and luciferase was quantified (Steady-Glo luciferase assay, Promega; Envision 96-well plate reader, Perkin Elmer).

### Animal studies

Experiments were conducted using female Balb/c mice (Charles River) aged 8–12 weeks in accordance with the United Kingdom Home Office Animals (Scientific Procedures) Act 1986 and in accordance with EU Directive EU 86/609. Colorectal CT26 tumors (ATCC) were established by subcutaneous injection of 5 × 10^5^ cells in 100 μL PBS into the flank. Animal weights were between 18 g and 23 g on the day of implantation. Tumor growth was monitored using callipers, and volumes estimated as half the product of the length multiplied by the width squared.

### TNFR2 expression profiling in tumor-bearing animals

10–12 days after tumor implantation, animals were sacrificed, spleens and tumors were removed. Spleens were disaggregated and filtered, and red blood cell lysis was performed (Sigma). Tumors were disaggregated according to manufacturer's instructions (mouse tumor dissociation kit II, GentleMACS dissociator, Miltenyi Biotech). Cells were stained using amine-reactive dye (Fixable blue dead cell stain kit, Invitrogen), washed and stained with panels incorporating CD45-BV570 (clone 30-F11), CD3-V450 (clone 17A2), CD4-APC (clone RM4.5), CD8-APC-H7 (clone 53–6.7), NKp46-EFluor450 (clone 29A1.4), CD11b-APC (clone RM4.5), Gr1-APC-Cy7 (clone RB6-8C5), and TNFR2-DyLight488 (clone TR75-89), or isotype-matched control mAbs. Subsequent flow cytometry analysis was performed as for human cells except mouse-reactive Foxp3-PE mAbs were used (clone FJK-16S).

### Investigating effects of TNFR2 mAbs *in vivo*

Eleven days after CT26 tumor implantation, animals were assigned to treatment groups by spiral randomisation such that all groups received an equivalent distribution of tumor sizes. On days 11, 14 and 18 after implantation, animals were injected i.p. with 100 μg of anti-TNFR2 mAbs (clones TR75-54.7 or TR75-89, Biolegend) or irrelevant control mAbs (clone HTK888, Biolegend) in 100 μL PBS. Animals were sacrificed when tumors reached maximum permissible size (15 mm average diameter). Animals in which tumors regressed completely were re-implanted with CT26 in the contralateral flank at least 31 days after tumors were last detectable. For flow cytometry analysis, animals did not receive the third dose of mAbs on day 18, and were instead sacrificed and organs processed as above. Cells were stained and analysed by flow cytometry as above except omitting TNFR2 staining. For cytokine analysis, 10^6^ splenocytes per well were added to wells of 96-well plates (Costar) in a volume of 200 μL RPMI1640 (Gibco) supplemented with 10% FBS, β-mercaptoethanol (500 μM; Life Technologies), PMA (20 ng/mL; Sigma), ionomycin (1 μg/mL; Sigma) and brefeldin-A (3 μg/mL, Ebioscience) and incubated at 37°C/5% CO_2_ for 5 h, after which they were washed, stained with CD3-BV650 (clone 17A2), CD4-BV785 (clone RM4-5), CD8-PerCP-Cy5.5 (clone 53–6.7) and CD19-APC-H7 (clone 1D3) mAbs, fixed/permeabilised, stained with Foxp3-PacificBlue (clone MF1-4) and IFN-γ-APC (clone XMG1-2) mAbs, and analysed by flow cytometry as above.

### Human lung tumor analysis

Surgical resection samples from three lung cancer patients were analysed by Caprion Biosciences Inc (Québec, Canada). This work was approved by the Comité éthique en recherche du Centre Hospitalier de l'Université de Montréal. Informed consent was obtained using informed consent forms. Tumors from patients 1 and 2 were adenocarcinoma (non-small cell lung cancer; NSCLC) and the tumor from Patient 3 was small cell neuroendocrine carcinoma (small cell lung cancer, SCLC). Briefly, tumor samples were cut into pieces using a scalpel, disaggregated mechanically (Medimachine, BD Biosciences) and stained using a panel including a dead cell detection reagent (Invitrogen), CD19-BV711 (clone HIB19), CD3-BV786 (clone SP34-2), CD4-BV605 (clone RPA-T4), CD8-BV650 (clone RPA-T8), TNFR2-Alexa647 (clone hTNFR-M1), GITR-PerCP-eFluor710 (clone eBioAITR), OX40-PE-CF594 (clone ACT35) or appropriate isotype-matched controls, and Foxp3-Alexa700 (clone PCH101) mAbs or appropriate isotype-matched controls. Samples were analysed by flow cytometry (Fortessa, BD Biosciences), and data analysis was performed using FlowJo X.0.7 (TreeStar).

### Cell-lines

All cell-lines used here were confirmed to be free of mycoplasma contamination using PCR, indirect Höchst stain and culture isolation methods (ECACC). Cell-lines were authenticated by short tandem repeat profiling, cell morphology, karyotyping, and cytochrome C oxidase I methods by the supplier (ATTC), and passaged for less than six months after resuscitation.

## SUPPLEMENTARY MATERIALS METHODS, FIGURES AND TABLE


