# Supplementary material for: Phenotypic screening reveals TNFR2 as a promising target for cancer immunotherapy
Source: Oncotarget. 2016 Sep 10;7(42):68278–91. doi: 10.18632/oncotarget.11943 (PMC5356554; doi:10.18632/oncotarget.11943)
Supplement: Supplementary file 1 [file oncotarget-07-68278-s001.pdf]

Phenotypic screening reveals TNFR2 as a promising target for cancer immunotherapy

Supplementary Materials

SUPPLEMENTARY MATERIALS AND METHODS

Cloning and expression of human TNFR2 constructs

Human *TNFR2* extracellular domain (ECD) fragments (Accession Number P20333) corresponding to residues 1–119, 1–205 and 1–257 were sub-cloned into a mammalian transient expression vector, upstream of a FLAG-His_{10} tag, and expressed in HEK293 cells. Purification from culture media supernatants was performed by affinity chromatography followed by size exclusion chromatography upon a HiLoad 16/60 Superdex 75 prep grade column (GE Healthcare, Bucks, UK).

Treg suppression assays

Following in vitro activation/expansion, Treg cells were labelled with PKH-26 (Sigma) and autologous Teff were labelled with CFSE (Invitrogen). 10^5 Teff cells per well were added to anti-CD3-coated 96-well U-bottom TC plates, together with anti-CD28 mAbs and 2.5 U/ml IL-2, and varying numbers of Treg cells, in a total volume of 200 µl per well X-Vivo medium supplemented with 5% human AB serum, and incubated for five-six days at 37°C/5% CO_2 in a humidified incubator. Finally, cells were analysed by flow cytometry (FACSCantoII, BD Biosciences), and Teff cell proliferation was assessed by CFSE dilution (proliferation tool, FlowJo, TreeStar).

TNFR2 expression by activated human lymphocytes

Cryopreserved PBMCs were defrosted and incubated in RPMI1640-based media supplemented where appropriate with 20 µg/mL PHA-P (Sigma) and 100 IU/mL IL-2 (Roche) for four days at 37°C/5% CO_2. Cells were re-suspended, washed once with flow cytometry buffer, stained using mAbs directed against CD3-APC-H7 (clone SK7), CD4-PerCP-Cy5.5 (clone RPA-T4), CD8-BV711 (clone RPA-T8), CD25-APC (clone BC96), CD56-PE-Cy7 (clone CMS5B), and TNFR2-FITC (clone 22235). Staining with Foxp3-PE mAb (clone PCH101) was performed according to manufacturer's instructions (Foxp3 fixation/permeabilisation kit, Ebioscience). Alternatively, PBMCs from HLA-A-positive donors with pre-defined reactivity to CMV pp65 (CTL) were defrosted, washed, and incubated in RPMI1640-based media supplemented where appropriate with 3 nM CMV peptide NLVPMVATV (Sigma) for five days. Staining with HLA-A/NLVPMVATV pentamers was performed according to manufacturer's instructions (ProImmune). Cells were finally washed, fixed and analysed by flow cytometry (FACSCantoII, BD Biosciences).

TNFR2 signalling assays

Jurkat E6.1 or HEK293 cells (ATCC) were transduced using replication-deficient lentivirus to express firefly luciferase via an NF-κB-responsive promoter, and further transfected using lipofectamine 2000 (Invitrogen) with a pcDNA3.1-based construct into which cDNA encoding human or mouse TNFR2 (NCBI sequences NM_001066.2 or NM_011610.3) flanked by 5′ GCCACC and 3′ TAATAA was inserted using BamHI and NotI (GeneArt, Invitrogen). Cells were selected for puromycin and geneticin resistance, cloned by limiting dilution or sorted for TNFR2 expression by flow cytometry. HEK293-based reporter assays were performed as for Jurkat-based assays, except using 3×10^4 HEK293 cells per well, and where appropriate 10 µg/mL goat anti-Armenian hamster IgG (Jackson Immunoresearch) was added to cells.

Analysis of publicly available RNAseq data

Publicly available RNAseq data (The Cancer Genome Atlas; http://cancergenome.nih.gov) were exported using Array Viewer (Omicsoft). For each gene analysed, fragments per kilobase squared (FPKM) values were summed for all transcripts, added to 0.1, and logarithmically transformed (base 2). Linear regression was performed using Prism 6.03 (GraphPad).

Measuring interactions between mAbs and mouse FcγRs

Surface plasmon resonance (SPR) measurements were performed on a Biacore T100 biosensor (GE Healthcare) at 25°C. Of note, ~3000 resonance units (RU) of monoclonal anti-His antibody (GE Healthcare, 28-9950-56) were immobilized on the CM3 surface by amine coupling (BIAapplications Handbook, GE Healthcare). The first flow cell served as a reference following one activation/deactivation cycle. The running buffer was HBS-EP (10 mM HEPES, 150 mM NaCl, 3 mM EDTA, 0.005% P20). Low-density surfaces

with ≤100 RU of His-tagged mouse Fcγ receptors I-IV (FCGR1, FCGR2B, FCGR3, FCGR4) were generated to minimize mass transport. Doubling dilutions of mouse IgG1 and IgG2a isotype controls or TR75-54.7/ TR75-89 anti-TNFR2 (Biolegend) in HBS-EP in the concentration range 10KD to 0.1KD were injected over affinity-captured Fcγ receptors at 40 μL/min. Surfaces were regenerated with a 30 second pulse of 10 mM glycine, pH 1.5. All sensorgrams were double referenced. Biaevaluation 4.1 software (GE Healthcare) was used to derive kinetic parameters by fitting the data with a simple 1:1 binding model or to obtain the dissociation constant for fast interactions through the equilibrium binding model.

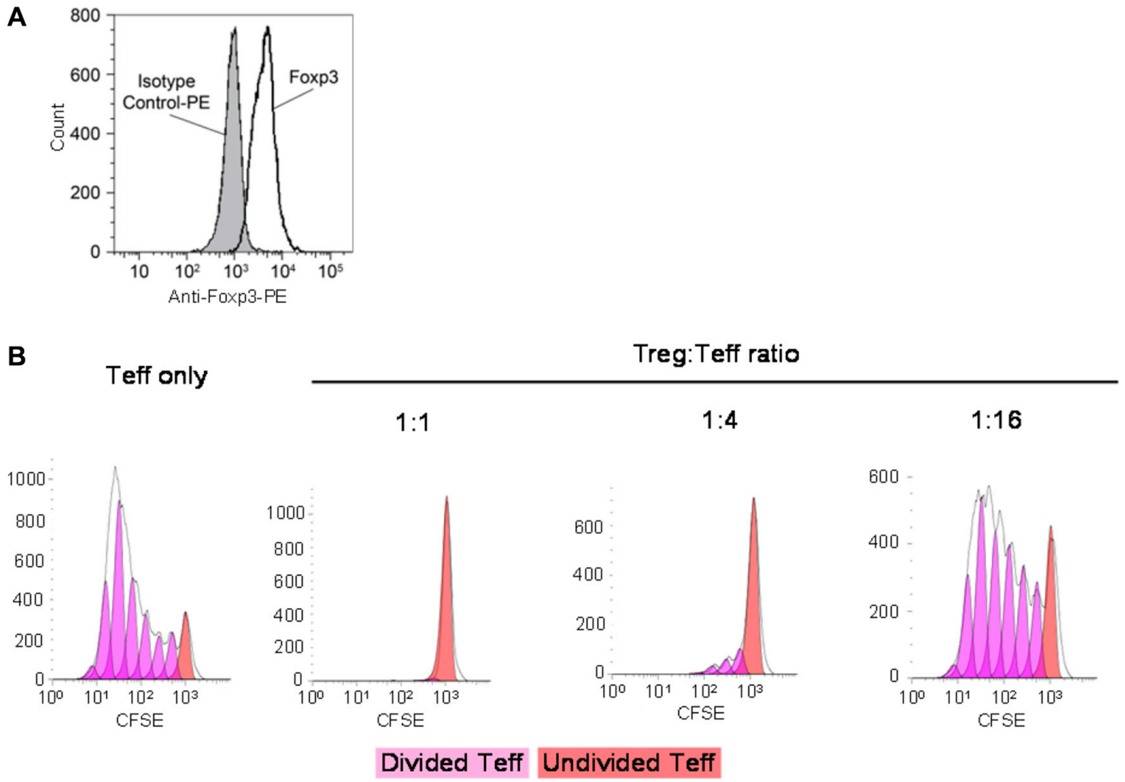

Supplementary Figure S1: High purity human Treg cell populations were expanded *in vitro*. (**A**) $CD4^{+}CD25^{+}$ Treg cells were isolated from healthy donor PBMCs by negative selection followed by positive selection for CD25 expression. Treg cells were activated and expanded using anti-CD3/CD28 beads and IL-2 in the presence of rapamycin. Purity was assessed using flow cytometry by staining fixed/permeabilised cells for the Treg-specific transcription factor Foxp3. (**B**) Following expansion, activated/expanded Treg cells were labelled with PKH-26 and co-cultured with CFSE-labelled autologous Teff cells in the presence of CD3/CD28 mAbs and IL-2 for five days. Teff cell proliferation was assessed by CFSE dilution. Data representative of Treg and Teff from seven independent donors.

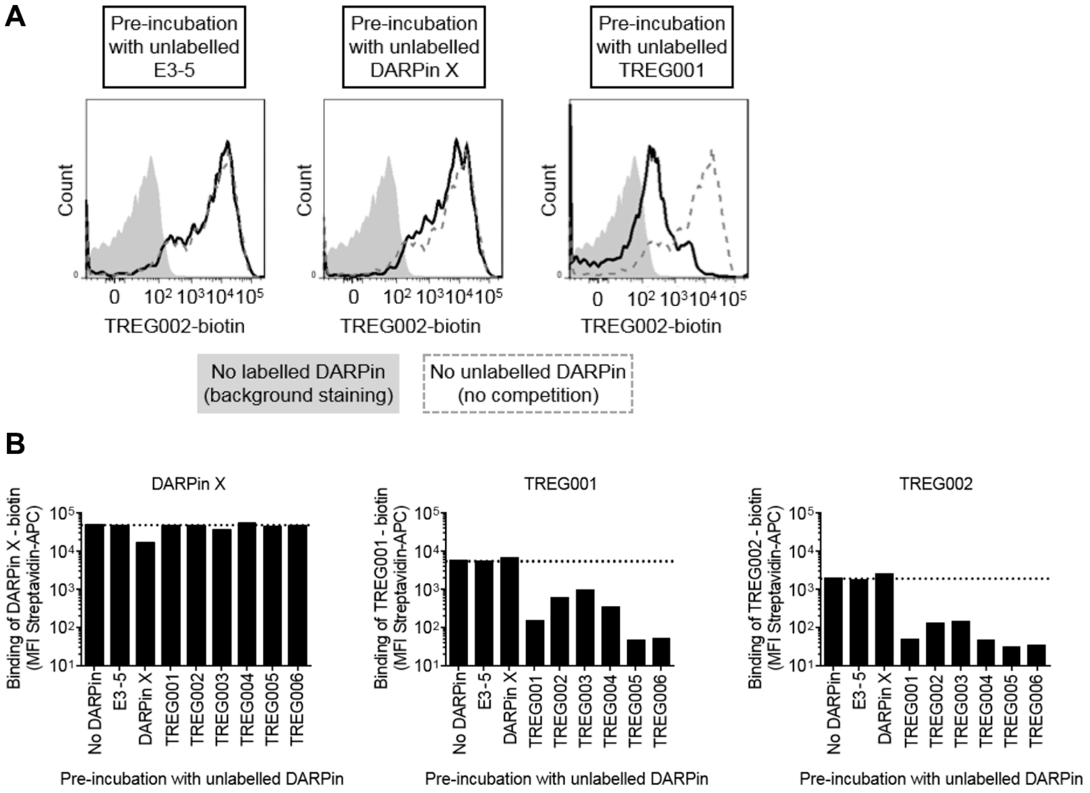

Supplementary Figure S2: Epitope redundancy amongst Treg cell-binding DARPins. (**A**) Activated Treg cells were pre-incubated with negative control DARPin E3-5 (which does not to bind to Treg cells), control DARPin X (which binds to all T cells), or each of the Treg cell-binding DARPins (data shown for TREG001). Subsequent binding of biotinylated TREG002 was detected using APC-labelled streptavidin and flow cytometry. (**B**) Summary data for Treg cells pre-incubated with TREG001-006 or control DARPins, and subsequently stained with biotinylated DARPins X, TREG001 or TREG002.

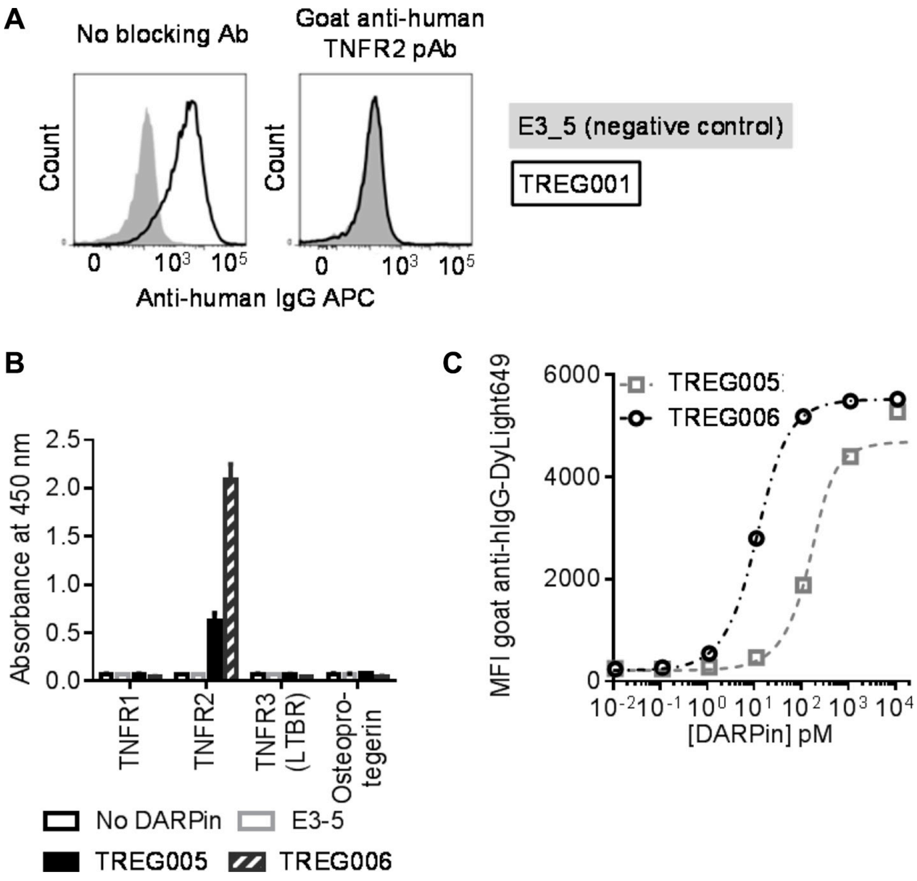

Supplementary Figure S3: Characterising binding between DARPins and TNFR2. (**A**) Activated Treg cells were incubated with or without polyclonal goat anti-TNFR2 Abs before staining with DARPins and APC-labelled goat anti-human IgG. (**B**) The ability of the DARPins to bind to TNFR1, TNFR2, TNFR3 (LTBR) or osteoprotegerin was investigated by ELISA. (**C**) TREG005 and TREG006 were titrated for binding activated Treg cells.

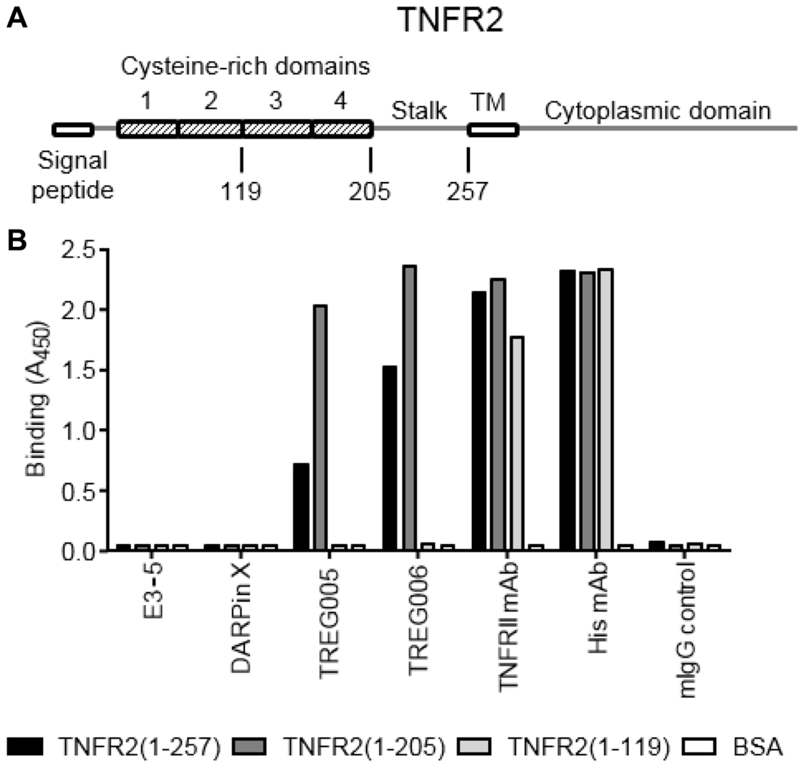

Supplementary Figure S4: DARPins bind to an epitope in the third and/or fourth cysteine-rich extracellular domain of TNFR2. (**A**) Structural schematic of TNFR2. Constructs comprising TNFR2 residues 1-119, 1-205 or 1-257 were expressed as C-terminal Flag-His_{10}-tagged fusions in HEK293 cells. (**B**) Binding of TREG005 and TREG006 to TNFR2(1-205), TNFR2(1-257) or TNFR2(1-119) was investigated using ELISA.

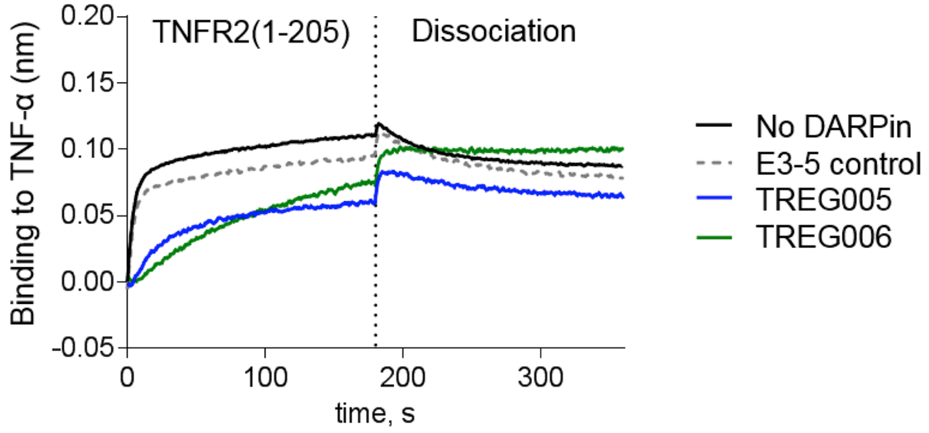

Supplementary Figure S5: DARPins bind to TNFR2 in a TNF-α-competitive manner. Binding of 150 nM recombinant TNFR2(1-205) to immobilised biotinylated TNF-α captured on streptavidin biosensors was investigated in the presence or absence of a molar excess (1.5 μM) of TREG005 or TREG006, or control DARPin E3-5.

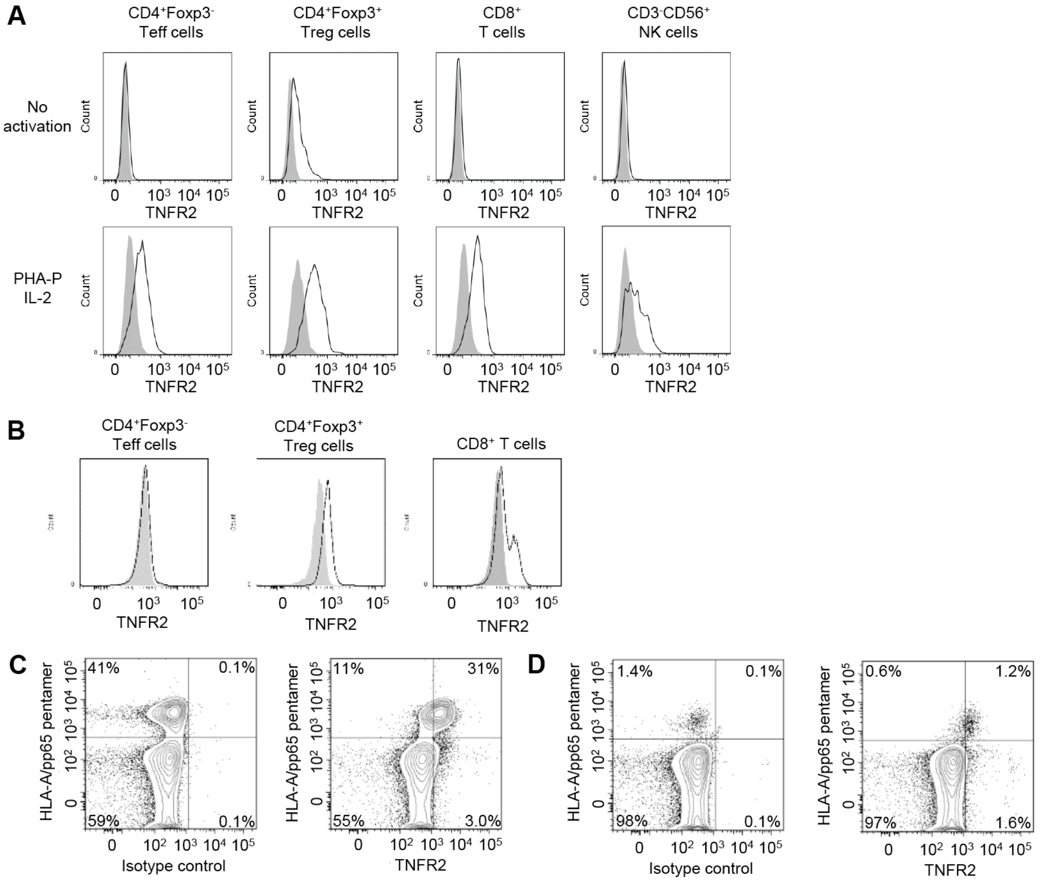

Supplementary Figure S6: TNFR2 is expressed upon T cell activation. (**A**) Human PBMCs were cultured for four days in the presence or absence of 20 μg/mL PHA-P and 100 IU/mL IL-2, and analysed by flow cytometry. Black lines indicate staining using anti-TNFR2 mAbs, while gray shaded regions indicate staining using isotype-matched negative control mAbs. (**B**) PBMCs from a healthy donor with pre-defined CMV reactivity were pulsed with 3 nM CMV pp65 peptide NLVPMVATV for five days and analysed by flow cytometry. (**C**, **D**) PBMCs from CMV-reactive donors with (C) greater or (**D**) smaller populations of pp65-specific $CD8^+$ T cells were pulsed with NLVPMVATV and stained using HLA pentamers to detect CMV pp65-specific T cells. Data for $CD8^+$ T cells are plotted. (B) and (C) were generated using PBMCs from the same donor, while (A) and (D) were from independent donors.

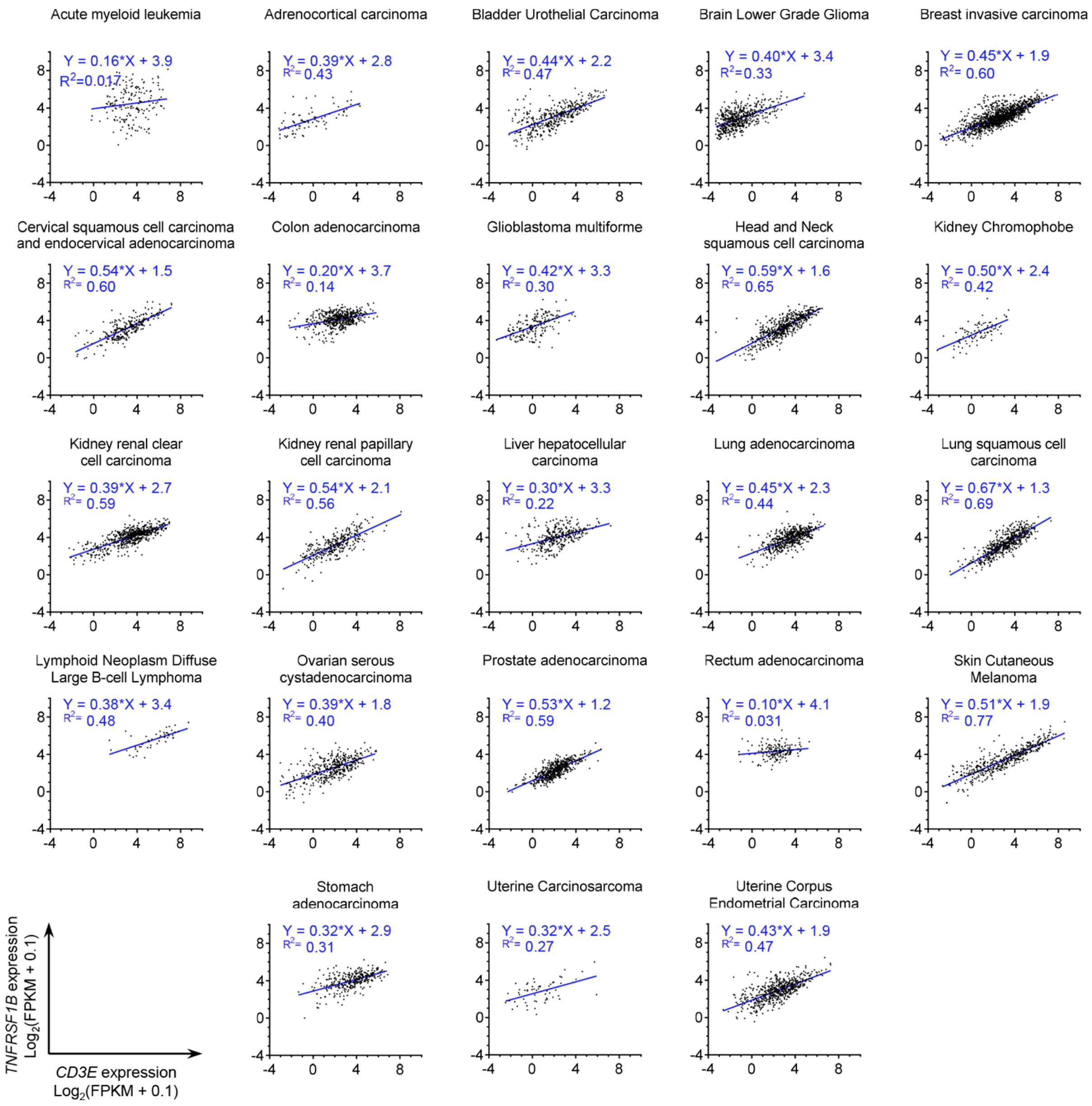

Supplementary Figure S7: *TNFRSF1B* expression correlates with *CD3E* expression for multiple tumor-types. RNAseq data for *CD3E* and *TNFRSF1B* were analysed by summing fragments per kilobase of exon per million fragments mapped (FPKM) values for multiple transcripts. Lines of best-fit were generated by linear regression. The results shown here are in whole based upon data generated by The Cancer Genome Atlas (TCGA) Research Network: http://cancergenome.nih.gov/.

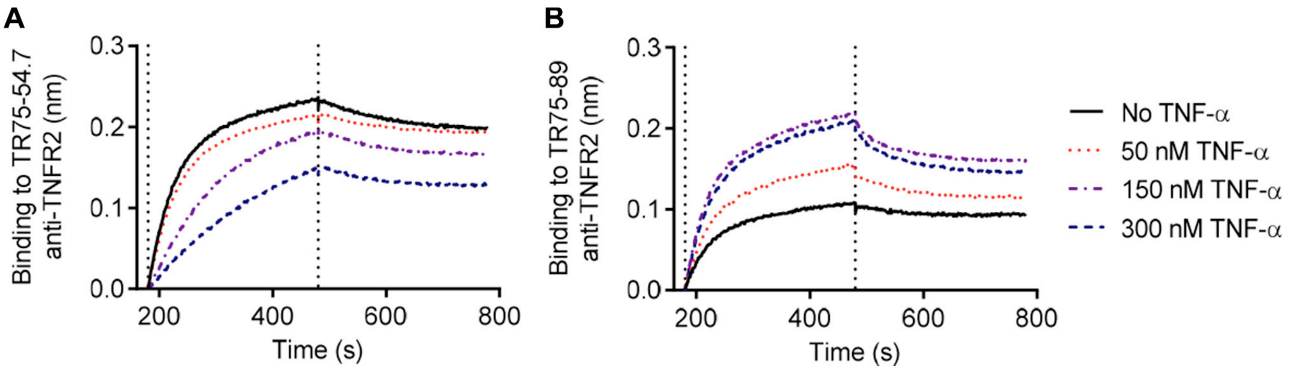

Supplementary Figure S8: TR75-54.7 mAb competes with TNF-α for binding to mouse TNFR2, while TR75-89 does not compete with TNF-α. 100 nM recombinant mouse TNFR2 was incubated with or without TNF-α, followed by assessment of binding to immobilised biotin-labelled anti-TNFR2 mAb (**A**) TR75-54.7 or (**B**) TR75-89 using streptavidin-coated biolayer interferometry sensors. Vertical dotted lines indicate transition from association to disassociation phases. In the case of TR75-89 (B), the enhanced signal magnitude observed in the presence of TNF-α suggests binding to a higher molecular weight species such as a TNFR2 ECD:TNF-α complex. The stoichiometry of the complex cannot be determined by this method, but it is possible that TNF-α-mediates multimerisation of TNFR2 prior to binding to immobilised TR75-89.

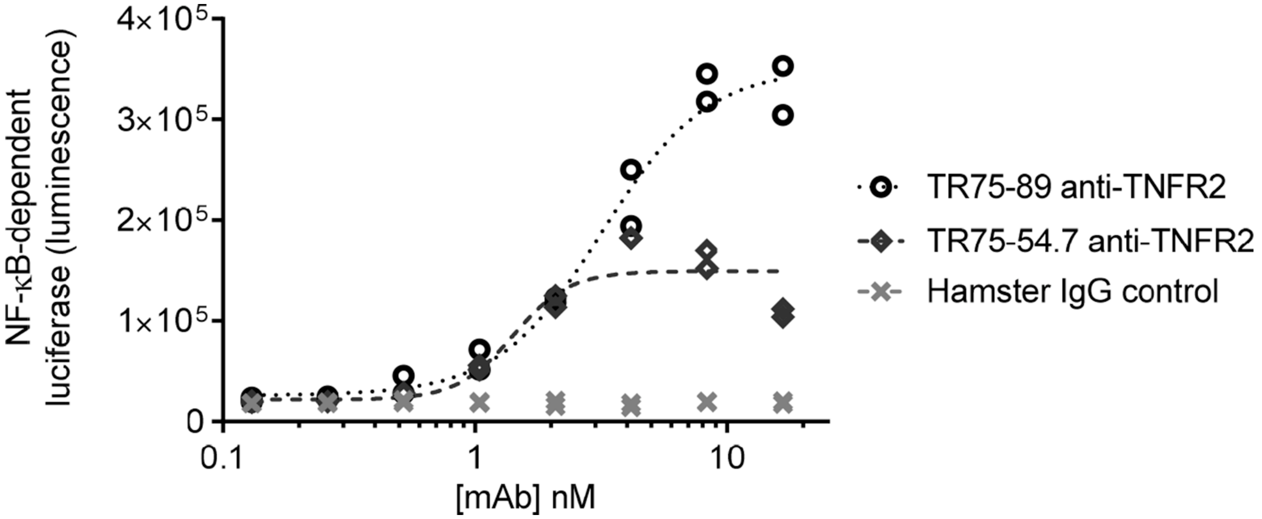

Supplementary Figure S9: Hamster anti-mouse TNFR2 mAbs are TNFR2 agonists *in vitro*. HEK293 cells were transfected to express mouse TNFR2 and NF-κB-responsive luciferase. Transfectant cells were incubated with hamster anti-TNFR2 mAbs TR75-54.7, TR75-89, or control hamster IgG, in the presence of 10 µg/mL goat anti-hamster IgG pAb for 5.5 hrs, after which luciferase expression was assessed by luminescence. Representative of three independent experiments.

Supplementary Table S1: Affinities of mouse and hamster mAbs for binding to mouse Fcγ receptors

mAb	Affinity of mAb for binding recombinant Fcγ receptor (K_D).			
	**FcγRI**	**FcγRII**	**FcγRIII**	**FcγRIV**
Mouse IgG_1 control	33 nM	1.5 µM	545 nM	40 nM
Mouse IgG_{2a} control	5.75 nM	2.11 µM	642 nM	9.45 nM
TR75-54.7 anti-TNFR2	Not measurable	615 nM	438 nM	Not measurable
TR75-89 anti-TNFR2	Not measurable	1.5 nM	3.2 nM	Not measurable

To investigate binding of hamster IgG anti-mouse TNFR2 mAbs TR75-54.7 and TR75-89 to mouse Fcγ receptors, surface plasmon resonance was used. His-tagged recombinant FcγRs were captured using biacore sensors coated with anti-His mAbs, after which control mAbs expressed as mouse IgG_1 or IgG_{2a}, or hamster mAbs, were flowed over the sensor.
